# How the Future Fell from Grace and How to Repair It. Changes in Time-Consciousness in the Late Twentieth and Early Twenty-first Century: a Response to Joe Davidson’s “From the Future to the Past (and Back Again?): a Review of Aleida Assmann’s Is Time Out of Joint? On the Rise and Fall of the Modern Time Regime (Ithaca: Cornell University Press and Cornell University Library, 2020)”

**DOI:** 10.1007/s10767-021-09413-8

**Published:** 2021-10-13

**Authors:** Aleida Assmann

**Affiliations:** University of Konstanz, Konstanz, Germany

## The Context and Genesis of the Book

The book that is being discussed in this generous format first appeared in German in 2013. It was well received but it did not have a longer impact. The question it posed was too unconventional and did not quite fit into the standard frames of discussion. But translated by the inspiring colleague and scholar Sarah Clift and accepted by Uwe Hohendahl in his remarkable book series ‘signale|TRANSFER: German Thought in Translation’, it has now the chance of a second life. Publication in English is a huge barrier for German texts, so I am most grateful to Clift and Hohendahl who made the book available to an English readership. I am deeply indebted to Joe Davidson who very carefully read and reassessed the arguments of the book and started a debate that updates these ideas and takes them into the future.

Davidson starts his essay with references to the retro-culture of the 1990s. I myself can testify to this shift with evidence from my own family household. I could indeed watch (with delight) my own children grow up with a fascination of and dedication to the very same pop songs that I had cherished as a teenager. The Beetles, the Rolling Stones and the Monkeys had been archived and canonised and were recycled 20 years later with the same lure and effect for a next generation who chose this particular music over the new offers manufactured and catered for their generation. Listening to the pop music of the 1960s was their personal choice and investment; buying into the retro-sound distinguished themselves from their peers.

This happened within a much larger context. The 1990s were the time of retro-culture. Raphael Samuel’s *Theatres of Memory* appeared in 1994, an influential book that captured this spirit by opening up a new historical access to unofficial worlds of the past that historians had hitherto ignored or detested. The book managed to establish ‘memory’ as a serious category for professional investigation, a term that hitherto stood for the opposite of academic ‘history’ and a legitimate and methodically sound approach to the past. Samuel’s book outlined a new space for serious empirical research between two positions that were at the time utterly irreconcilable: conservative politicians of the Tory-Thatcher government who promoted national pride, patriotism and a revival of Victorian values, and, at the other end of the spectrum: left historians who fervently opposed concepts like identity, national nostalgia and what they called the ‘heritage industry’.^1^

Raphael Samuel was a historian who discovered that a divide was opening between more and more austere professional approaches to the past on the one hand and new unprofessional accesses to the past as ‘public history’, energized by family history, psychological transmission, social imaginations and cultural stagings on the other. Rather than dismissing and delegitimizing this new development as groundless fiction and kitsch, Samuel registered a historical shift and introduced new concepts for dealing with it within a new critical framework. Accepting and adopting the controversial term ‘memory’ for this shift was an important strategic move. He showed that retro-culture was no longer to be conceived as a purely regressive and backward-looking phenomenon (which of course it can be when fueling social nostalgia and reactionary nationalism), but rather in terms of a more deeply human enterprise and general symptom of exploring new subjective ways of relating to time and dealing with the past.

I would like to add here another movement that was effectively pushing a new temporal orientation between the 1980s and 2000, this time not from the point of view of historians and sociologists but from my point of view as a scholar of literature, the arts and media. The ‘archive’ for instance was an important concept that transcended its place and meaning in concrete cultural institutions and became a much broader metaphor for artistic understanding, practices and trajectories.^2^ These have to do with conscious recoveries of fragments and traces of a latent or buried past that had long been ignored, neglected or covered up. Together with the terms ‘archive’ and ‘traces’, other terms like ‘trauma’ and ‘testimony’ moved centre stage and became seminal concepts for new approaches to history or better: his- and herstories.

‘Recycling’ is a further key term that appeared in the world of the arts and reflected a shift in the given cultural system of temporal ordering. Taken over from ecology and theories of sustainability, it had a remarkable career in the field of artistic ‘creation-through-transformation-of-a-previous-existing-text’. The U.S.-American writer Michael Cunningham, for instance, based his novel *The Hours* (1998) on the first draft of Virginia Woolf’s *Mrs Dalloway* (1925). His text is organized as a transparent structure that keeps the ‘pre-text’ visible, creating specific resonances between the psychic depression of Woolf in the 1920s and the aids pandemic in New York in the 1990s. This type of art aims at a new form of innovation that is no longer a tabula rasa but aims at a charged conjunction and dialogue between different historical periods.

Joe Davidson begins his review with various post-modern returns to the past: retro-culture, nostalgia and melancholia. Influential and important as they were, he is definitely right that these phenomena are not at the centre of what triggered the key questions of my book. My key question was: What time regime did we leave and give up when we moved on to these new involvements with various pasts? What had been the normative sense of time in the world into which I grew up, the world of the Cold War? I called it the ‘modern time regime’ and tentatively dated it from 1945 to 1990, the reunification of Germany. This task was both a personal and academic exploration; it required a broad collection and interpretation of textual sources which did not exist in the compact shape and package of a ‘discourse’, a ‘period’ like the British discussion on history and memory referred to in the first footnote of this essay.

In addition, it required a self-analysis and -interpretation, because I had lived in this time regime and became its witness. In the 1980s and 1990s, when England coped with the process of decolonizing its empire and experimented with a new national past and heritage industry to boost patriotic pride, Germany was in a very different situation. May 8, 1985, is the date of a historic speech by president Richard Weizsäcker commemorating the 40th anniversary of the end of the Second World War. In this speech, he persuaded the Germans to interpret this date no longer in the light of a general and absolute defeat but to accept the idea of liberation as a new perspective for the national narrative. In 1986, one year after this offer form above to share the European narrative of the neighboring countries, the historian’s debate took place that established the singularity of the Holocaust as a historically unprecedented and incomparable event. It provided a second step in the direction of what would be later called Germany’s ‘memory culture’, namely embracing the Holocaust as a central and defining national memory.

The collaborative work on cultural memory that Jan Assmann and I developed in our joint research started in these years. Our defining background was not the Zeitgeist of retro-culture but the belated formation of a cultural memory of the Holocaust four decades after the end of the War and its stabilisation in a transnational memory community. Synchronous with the establishing of this memory, we developed our theory of cultural memory with this very special Holocaust memory formation before our eyes. This is also the point of departure, where my time project started: I wanted to find out retrospectively in what a time formation I had lived that for four decades had prevented the possibility of forming such a memory. I found out that the repression of the Nazi past in Germany took place in a much wider culture of forgetting shared by Western (and Eastern) countries, where the forgetting of the past was issued from a shared normative framework of modernisation. These are some of the questions that propelled my research: What is the advantage of such a time regime? Why should the past be suppressed? Who profits from suppressing the past, and who profits from remembering the past?

My task was clear: I wanted to spell out as distinctly and clearly as possible the major principles that made up the modern time regime. As they had been firmly and safely embedded in institutions, embodied in habits and embedded in frames of thinking, they had remained implicit and enjoyed what Niklas Luhmann has called ‘Latenzschutz’, the protection of latency. Such a task had never been undertaken, which is the reason that I had to collect my sources in various fields. The recovery of the five implicit principles of the modern time regime was made possible with the waning of its power and persuasiveness, and it was this waning that allowed us to embark on the topic of cultural memory. To this day, the principles of the modern time regime and the principles allowing us to study cultural memory persist and present a clear case of cognitive dissonance. The irreconcilability of both frames of thinking is ongoing. In the humanities, the study of cultural or collective memory remains a scandal for those who continue to pursue the path of ‘modernisation theory’. They only accept the concept of an individual memory and reject everything else as an illegitimate fantasy. An illustrious German intellectual wrote me a letter of thirteen narrowly printed pages in which he urged me to give up the false idea of memory as a connecting and binding force in the constitution and organization of groups. To this day, modernization theorists in the humanities vehemently oppose and suppress the idea of a shared memory as a possible methodology and field of study connecting various disciplines.

After having placed the theme of cultures of temporal ordering in its context and after having outlined my book’s intentions with incredible scrutiny, attention, care and brilliance, Davidson ends his essay with a generous assessment: ‘Is Time Out of Joint? is certainly not the last word on the modern time regime; its haunting presence in the cultural consciousness means that, for good or bad, we can never quite lay it to rest. (…) Instead, it represents the first word on temporality in the twenty-first century, offering a productive framework for addressing our disturbed and unsettled time consciousness.’ (12) Davidson is right: writing about the future should be inspired by a spirit of opening rather than closing the topic. In the meantime, I have continued to rethink issues of cultural temporality. This dialogue gives me a welcome chance to sum up the direction of my recent ideas on the subject.

## Three Futures

In these times of ecological crisis and global transformation, our thinking about the future takes place under the general suspicion that there is not much left of it. This explains why talking about the future today is hardly possible without adopting a melancholic or apocalyptic tone. I will guard against this with two simple strategies. Firstly, I do not ask whether we ‘have or do not have’ a future, but how we ‘make’ a future. This can also be called ‘doing future’. ‘The’ future is never an objective entity, it is created by doing something for it and investing in it: planting an apple tree, giving birth to a child, creating a work of art. Secondly, there is not just one ‘future’ that is practised and imagined in our society. When moving from the singular to the plural, the next question is: Which futures? In the following, I will briefly sketch three meanings of future that are currently circulating. They are known to everyone but never invoked together because they compete with and contradict each other. Nevertheless, they are all in use and we need them all. By broadening our scope of perception, it is to be hoped, we can also broaden the scope of our thinking and distance us from mono-mythical visions of the future.

## First Meaning: Rupture, Change, Renewal, Progress

My first meaning of future returns once more to the modern time regime (MTR) that is expressed in terms such as rupture, change, renewal, innovation and progress. They are all deeply anchored in the culture of modernisation and its narrative of progress. The Western world has produced the modernisation process and the modernisation process has produced the Western world, so the West and the idea of progress are mutually reinforcing.^3^ In this view, future and past have nothing to do with each other. They are apodictically opposed to each other as the ‘old’ and the ‘new’, whereby the old is synonymous with ‘past’ and ‘obsolete’, while all hopes are directed towards the new. In order to be fully attuned to a better and brighter future, one must shed the past and forget it. During my youth and student days, this unbridled optimism about the future prevailed in post-war West Germany. Many people quickly achieved status and prosperity after the ravages of the Second World War. Modernisation became a central concern in the cities, as old rubble was extended by new rubble: many historic buildings that had survived the war were removed and replaced by new buildings. Brand new technical inventions such as television, concrete and plastic changed the world we live in. In 1969, mankind’s dream of landing on the moon was realised. But ten years later, this upward trend flagged. In 1979, Jean-Francois Lyotard registered the ‘end of grand narratives’, by which he meant above all the modernist narrative of progress. With the end of the Cold War, the MRT lost its lure as a binding cultural programme.

But, this was by no means the end of it. Science, technology and economics remain the driving forces of modernisation and, whether we like it or not, continue to form the backbone of Western civilisation. There is no easy exit from modernisation as we remain dependent on it and need new technology to preserve the environment and to regain the future. The age of mobilising ideologies may be over, but the narrative of innovation and progress remains in force in the universities, in economics and technology. In some domains, the conditions for innovation have become even tougher, for instance, in Silicon Valley where new models of ‘disruptive innovation’ erase and replace ‘evolutionary innovation’.

## Second Meaning: Uncertainty and Risk Management

My second meaning of future refers to everything that is unknown, unexpected and as yet unthought of. This unknown future comes to us as a pleasant or unpleasant surprise. “Que sera, sera”—some of us still remember the words of Doris Day’s hit in the 1950s:

*Que sera, sera,*

*whatever will be, will be,*

*the future’s not ours to see,*

*que sera, sera.*

The idea of an uncertain future exists in all cultures of the world: the future is inevitably and precisely that which people cannot see. It is a view that is categorically withheld from humans and reserved to God, a humbling effect that is still echoed in idiomatic formulas like ‘insh’Allah’ or ‘deo volente’ that emphasise the fundamental inaccessibility of the future. The notion of the unknown future is often combined with a fatalistic attitude towards time. The wisdom of Doris Day’s song therefore consists in self-restraint: que sera, sera—we have to wait patiently and accept, for better or worse, what the future has in store for us.

But, there are also more active ways of responding to the unknown future and shielding us from total uncertainty. In Mesopotamia, there were elaborate techniques for gaining control over the enigma of the future. The Assyriologist Stefan Maul speaks of ‘Zukunftsbewältigung’, coping with the future, a term he formed in analogy with ‘Vergangenheitsbewältigung’. In the Middle Ages, the future was the domain of the unpredictable goddess Fortuna, who was said to be constant only in her change. In the early modern period, attitudes to the future changed fundamentally when concepts like ‘accident’ and powerlessness were replaced by concepts like ‘risk’ and ‘opportunity’. The first great entrepreneurs who set out across the ocean to the New World did so with a new compass and in a spirit of self-empowerment in the face of an uncertain future. Slogans like ‘sapere aude!’ (be brave to know) or ‘who dares will win!’ reaffirm trust in human action in the face of radical uncertainty.

The term risk is much broader than ‘accident’. Today, we no longer cope with the uncertainty of the future with the help of oracles and rites, but with opinion polls, the stock market and insurance companies. Future modelling techniques such as scenarios and diagrams help create security and continuity in the face of risk and uncertainty. This ensures a minimal certainty of action, even in times of a global pandemic, but it also has its limits. Neither the fall of the wall in 1989 nor the election of Donald Trump in 2016 had been anticipated, to say nothing of the COVID-19 pandemic in 2020!

## Third Meaning: Sustainability

My third meaning of the future was added in the 1970s with the rise of a new ecological consciousness. The writings of the Club of Rome spread the news about the knowledge of the limits to growth and the finiteness of natural resources. This caused a radical shift in our relationship to the world and our orientation in time. It also leads to an experience of loss that is captured in another pop song, this time in a refrain by Joni Mitchell:

*(that) you just don’t know what you’ve got*

*till its gone.*

We are indeed learning what we possess by a sudden awareness of loss. Nature, we now understand, is no longer the stable resource independent of humans that was fantasised throughout the ages. On the contrary: the truth that we have to face is that nature is permanently affected, changed and destroyed by humans. Likewise, the future that was once a projection screen for our hopes and desires has itself become an object of care and provision, of heightened attention and permanent responsibility. The future that had served the realisation of human desires and purposes can no longer be taken for granted or be relied upon. On the contrary, humans must make a joint effort in order to create a future for future generations.

In this third meaning of ‘sustainability’, the future has entirely shed the first meaning of rupture, change and innovation and now conversely refers to resources and species that already exist, which are cherished with the hope that they continue to exist. The word ‘sustainability’ originated in forestry, where it referred to the amount of trees that needed to be replanted after forests were cut down. In 1986, the word was redefined in the so-called Brundland Report: In the process, sustainability became the key term of a new field of research spanning various disciplines. Future in this sense also relies on change, but it is a second-order change that is meant to encompass and correct man-made change and its hitherto ignored and neglected collateral damage. This future also reckons with ruptures but they are no longer driven by a human power but by unintended consequences of human action such as climate change and the extinction of various species. Instead of valuing contingency or gambling with risk and chance, the sustainable future is focused on the preservation and continuity of what we already know, possess, use and value.

When, in August 2016, a group of experts announced the entry of mankind into the new geological age of the ‘Anthropocene’, this did not only dramatically expand the horizon into the past and the future, but also included a totally new notion of human responsibility. As humans, we now no longer live with a short- or medium-term view of the future, but find ourselves in a completely different historical narrative, a ‘big history’ that calculates backwards and forwards in millennia and millions of years, in which human existence is only a brief episode. In the light of the new big history narrative of the Anthropocene, it also becomes obvious that the destruction of Europe through wars and weapons in the first half of the twentieth century was seamlessly followed in the second half of the same century by another phase of destruction through modern science and technology, this time affecting the entire planet. In this perspective, the era of peace and progress in the 1950s and 1960s that followed the ravages of two World Wars worked irreversible havoc on the planet and endangered its future. The perspective of sustainability—Future 3—thus turns out to be the averted and unreflected flip side of modernisation thinking—Future 1.

Ecologic sustainability is connected to the Greek word ‘oikos’ meaning ‘household’ and thus refers to responsible and frugal housekeeping. Since 1996, a new way of modeling the future was presented as a temple of sustainability, the roof of which is upheld by the three pillars designating the economic, the ecological or environmental, and the social, otherwise also known as the three ps: people, planet and profit. The assumption is that these aspects have to work together to make long-term and sustainable development possible. The word ‘development’ in this sentence is deceptive, however, firstly because it continues the jargon of modernisation and secondly because it obscures the fact that these three dimensions are by no means congruent. Environmental sustainability assumes clear limits to growth, while economic sustainability continues to focus on profit and growth. Social sustainability, on the other hand, refers to a principle of class and generational justice that can only be implemented through a political turnaround. What is needed, in other words, is a new ecological global contract that legislates and coordinates the joint efforts of humanity to avoid further irreversible ‘developments’ for which there are no longer any solutions available.

Our language is tricky because sustainability is not only a good thing. We are confronted with a growing accumulation of things that we can no longer get rid of: Wealth waste, radioactive deposits and the sealing of soil by pollutants that are no longer degradable. These things will—as experts assure us—still leave their traces in the earth’s history millions of years from now. In the field of history, too, there exists also a negative form of sustainability in the form of historical traumas. Since time does not heal these wounds, traumatic violence produces a ‘past that does not fade away’ and which still needs reworking decades and centuries later. The negative sustainability of traumatic pasts had been ignored and suppressed for a long time. Here, too, a new concept of responsibility has emerged, this time within the political, social and cultural framework of a regime of human rights. This responsibility manifests itself in a subsequent processing of historical wounds through memory, recognition and empathy with the intention of neutralising the destructive sustainability of trauma and transforming it into (self-)critical provision. Such a past, writes Amir Eshel, “is always already of a double nature: as an unbearable burden, it simultaneously opens up a space of possibility for change—its ‘futureness’ is the potentiality of history, the appearance of the better and the measure of our actions in the here and now.”^4^

Where do we get from here? Davidson has a concrete advice. He himself has written copiously about a fourth form of future, the utopian future, which he introduces at the end of his essay. His advice is clear and pithy. He contends that ‘some of the concepts associated with the modern time regime – such as the desire for a revolutionary break and the need for radical utopian imaginaries – have, if anything, become more vital in recent years. (…) if we desire a viable future in which the environmental preconditions for human life are secured, then a radical break from prevailing social relations is required, and required quickly.’ (11) As every action starts in the minds and hearts of people, this requires a collective will, which, however, can only be inspired by a utopian spirit.

I like this leap of the imagination and support it with my own pleas. The first is to think about the future henceforth more in the plural: future as a promise of progress, as risk management, and as positive and negative sustainability in nature, culture and history. The fact that we have not yet brought these different meanings of ‘future’ together in a more complex concept is mainly due to the discursive borders between our disciplines. My second plea is to make the temple of sustainability more sustainable by adding a base to it in which the three pillars are anchored. The name of this base would have to be ‘culture’. Culture is a term that also exists only in the plural. What is in demand, therefore, is a diverse yet inclusive concept of culture that respects the demands of the planet and eschews values and practices that are contrary to it. Accelerated global and planetary change makes joint efforts increasingly urgent. Scholars concerned with issues of sustainability could be more agile in interweaving political, ecological, economic, technical and cultural perspectives in their respective discussions. This could be their substantial contribution to ‘doing future’.

## Footnotes

1 Here are a few book titles that reflect the tension between history and memory in the British discourse: Robert Hewison, *The Heritage Industry: Britain in a Climate of Decline,* London: Methuen 1983; Eric Hobsbawm, Hugh Trevor-Roper, eds., *The Invention of Traditions,* Cambridge: Cambridge University Press, 1983, Patrick Wright, *On Living in an Old Country*: *The National Past in Contemporary Britain*, London: Verso, 1985; David Cannadine, ‘Viewpoint British History: Past, Present – and Future?’, *P&P*, 116 (1987), 169–91; Raphael Samuel, Paul Thompson, eds., The Myths we live by, Routledge 1990; Raphael Samuel, *Theatres of Memory: Past and Present in Contemporary Culture,* London: Verso, 1994.

2 Aleida Assmann, chapter on ‚archives ‘ in *Cultural Memory and Western Civilization*; Diana Taylor, *The archive and the repertoire*; Kynaston McShine ed., *The Museum as Muse*, exhibition catalogue, Museum of Modern Art, New York, 1999.

3 It should be noted that the belief in the time regime of modernity is not confined to Western capitalism alone but includes Eastern communism. The break with and dismissal of the past, combined with an enthusiastic embrace of a future promising and delivering progress, was shared by both antagonists during the Cold War. The first line of the national anthem of the DDR succinctly summed up this condition: ‘Risen from the rubbles we are facing a bright future’. Both Russia and the USA trusted in scientific and technical progress and shared a vision of the future that would bring humans to the moon.

4 Amir Eshel, Futurity: Contemporary Literature and the Quest for the Past. The University of Chicago Press, 2013.

